# Symptoms of SARS-CoV-2 infection and vaccine status of sixty-seven adult patients affected by inherited metabolic diseases: a phone survey

**DOI:** 10.1186/s13023-023-02905-0

**Published:** 2023-09-12

**Authors:** Lucia Brodosi, Michele Stecchi, Dorina Mita, Francesca Marchignoli, Valeria Guarneri, Giulio Agnelli, Valentino Osti, Federica Perazza, Federica Sacilotto, Andrea Pession, Loris Pironi

**Affiliations:** 1 Department of Medical and Surgical Sciences, University of Bologna, Via Zamboni, 33, Bologna, 40126 Italy; 2 Clinical Nutriti on and Metabolism Unit, IRCCS AOUBO, Via Albertoni, 15, Bologna, 40138 Italy; 3 Department of Public Health, Azienda USL di Bologna, Via Gramsci, 12, Bologna, 40121 Italy; 4 Pediatric Unit, IRCCS AOUBO, Via Albertoni, 15, Bologna, 40138 Italy

**Keywords:** COVID-19, SARS-CoV-2, Vaccination, Inherited metabolic diseases, Hereditary metabolic disorders

## Abstract

**Background:**

The Covid pandemic seems to have had several detrimental effects on managing patients affected by inherited metabolic diseases (IMD), although published data about the impact of COVID-19 on patients suffering from IMD are very scarce. The scope of our work was to evaluate adherence to the vaccination plan, the side effects experienced by our adult IMD patients, and the symptoms of the SARS-CoV-2 infection.

**Results:**

Sixty-seven patients agreed to respond to a phone interview. The mean age was 36.5 (± 11.6 SD). Regarding the vaccination campaign, fifty-five patients (82%) joined it, of whom ten had received two doses and the remaining forty-five, three. Forty-two patients (76%) reported adverse events following vaccination, the most frequent being local reaction, fever, and asthenia, which lasted an average of two days and resolved without sequelae. Regarding SARS-CoV-2 infection, twenty-seven out of sixty-seven patients (40%) tested positive for the virus; seven of them were not vaccinated at the time of infection; on the other hand, twenty had already had at least two doses. Regarding the prevalence of long-Covid, as many as 12 patients (44%) reported symptoms that persisted after the nasopharyngeal swab tested negative and lasted an average of 81 (± 74 SD) days. There were no statistically significant differences in BMI of patients who contracted the infection and patients who did not (25.15 vs. 25.20, p = .861), between those who had adverse reactions to the vaccine and those who did not (24.40 vs. 25.75, p = .223), between those who had long-Covid and those who did not (25.9 vs. 27.7, p = .183). No relation was observed between metabolic inherited disease, SARS-CoV-2 infection symptoms and adverse vaccine reactions.

**Conclusions:**

The data indicate that IMD patients adhered to the vaccination campaign comparably to the general Italian population. Adverse events to the vaccine were negligible. SARS-CoV-2 infection, which occurred in most cases after receiving at least two doses of the vaccine, did not cause serious symptoms and never required hospitalisation. A non-negligible share of patients suffered from long-Covid symptoms.

## Introduction

The Covid pandemic seems to have had several detrimental effects on the management of patients affected by inherited metabolic diseases (IMD), although published data about the impact of COVID-19 on patients suffering from IMD are very scarce.

Most authors focused on the negative impact of lockdown on IMD patients’ management.

From a survey of The European Reference Network for Hereditary Metabolic Disorders (MetabERN), most patients experienced a disruption of care, with appointments cancelled or postponed. Almost all (90%) healthcare providers promptly substituted face-to-face visits with telemedicine. About half of the patients faced changes in treatment and switched from hospital to home therapy; in addition to this a quarter reported that getting medicines was harder [1].

A multicentric data collection from 16 specialised medical centres treating IMD patients in Europe, Asia and Africa confirmed the profound impact of the pandemic on patient management and care, with difficulties in visits, lab examinations, and instrumental assessment of patients [2].

A study evaluating Pompe disease showed similar results, the interruption of therapy due to the pandemic worsened patients’ motor and respiratory function [3].

It has also been reported that many healthcare units were obliged to switch endovenous therapy administration from hospital to home in order to guarantee therapeutic continuity during the lockdown, preventing the inevitable metabolic derangements that would have occurred otherwise [1, 4, 5].

From a psychological point of view, one article focused on patients affected by phenylketonuria (PKU) highlighting how the pandemic increased anxiety levels, moreover in female patients; the main concern reported was linked to the fear of not being able to obtain special nutritional products needed to keep the underlying IMD properly managed [6]. Despite the emotional impact, PKU patients’ metabolic control did not worsen [7].

The aforementioned studies delved into the management and psychological impact during COVID 19 pandemic, but we observed scarcity of studies evaluating the course of the disease in IMD patients and the impact of COVID-19 vaccination.

MetabERN promoted a critical survey among 78 healthcare centres from 23 EU Member States dealing with different IMD [8]. They reported 452 cases of SARS-CoV-2 infection among their patients, both children and adults, with a prevalence of 17%. Most paediatric cases of infection displayed either mild or no symptoms while infected; however, 3% of centres reported the death of patients who tested positive. Similarly, most adult infected patients were asymptomatic or had mild symptoms. Severe symptoms requiring hospitalisation and COVID-19-reported deaths in adult IMD patients were claimed respectively by 31% and 15.4% of respondents [6].

In addition, Altassan et al. reported that unvaccinated patients with IMD older than 16 presented with moderate to severe symptoms in 20.5% of cases [9].

On a Duch cohort of 169 Pompe patients, fifteen (8.9%) reported a SARS-CoV-2 infection. Except for one case of myocarditis (not requiring hospitalization), only mild side effects were reported, and no patients needed intensification of ventilatory support. The estimated prevalence of vaccination was 98% [10].

Given the scarcity of data in the literature, we wanted to evaluate adherence to the vaccination plan, the side effects experienced by our IMD patients, together with the symptoms of the SARS-CoV-2 infection.

## Results

Of the ninety-three patients we tried to contact, sixty-seven agreed to respond to our phone survey; more than half were affected by PKU or hyperphenylalaninemia (41/67, 61.2%). The mean age was 36.5 (± 11.6 SD), and the mean body mass index (BMI) was 25.5 (± 4.5 SD). The patients were equally distributed between males and females, and only a few were smokers. All characteristics recorded are shown in Table 1. All comorbidities were mild, without any relevant effect on day-to-day life. No female patients were pregnant either at the time of vaccination or infection.

**Table 1:** Sample characteristics

Characteristic	Overall, N = 67, n (%)
Gender	F 35 (52%), M 32 (48%)
Smoking	yes 8 (12%), no 59 (88%)
Metabolic Disease	
Phenylketonuria Glycogenosis Galactosemia Hyperphenylalaninemia Homocystinuria MTHFR Deficit Hereditary Fructose Intolerance Tyrosinemia type 1 Gyrated Atrophy of the Choroid Primary Carnitine Deficiency	38 (56.7%) 10 (14.9%) 4 (6%) 3 (4.5%) 3 (4.5%) 3 (4.5%) 3 (4.5%) 1 (1.5%) 1 (1.5%) 1 (1.5%)
Comorbidities	
Gastrointestinal Endocrinological Nephrological Cardiovascular Neurological Pneumological	16 (23.9%) 12 (17.9%) 8 (11.9%) 6 (8.9%) 5 (7.5%) 3 (4.5%)

BMI = Body Mass Index; MTHFR Methylenetetrahydrofolate reductase

All patients clearly remembered their vaccination status, the experienced adverse reaction to the vaccine(s), if they ever tested positive for SARS-CoV-2 and the disease course.

Regarding the vaccination campaign, fifty-five patients (82%) joined it, of whom ten have received two doses and the remaining forty-five three. In particular, 39 patients (70%) received homologous vaccination (same type of vaccine - mRNA), and 28 (30%) heterologous (vaccine with adenovirus plus mRNA vaccine(es)).

**Fig. 1:**
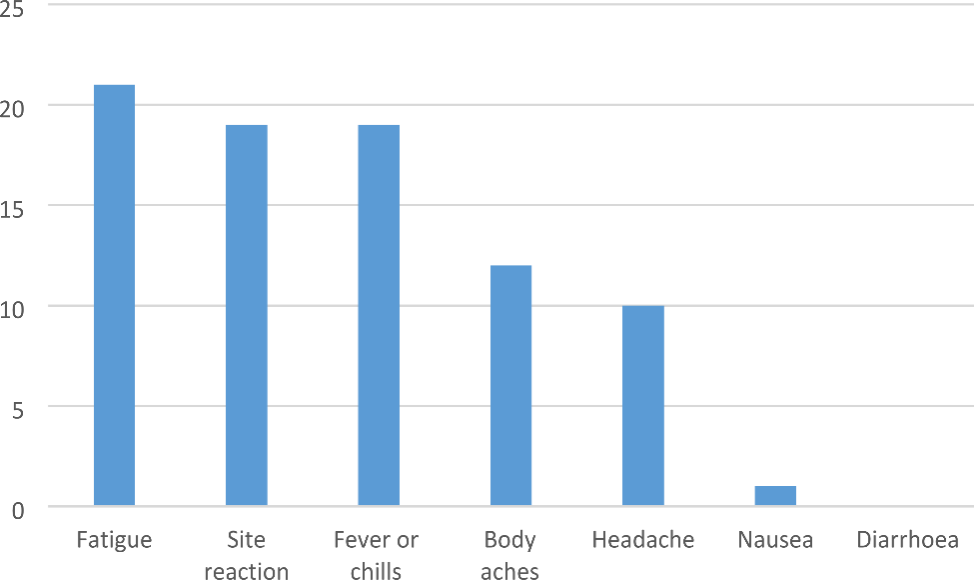
Frequency of adverse events after vaccination

Forty-two patients (76%) reported adverse events (AEs) following vaccination, the most frequent being local reaction, fever, and asthenia, which lasted an average of two days and resolved without sequelae. The reported AEs are shown in Fig. 1.

Regarding SARS-CoV-2 infection, twenty-seven out of sixty-seven patients (40%) tested positive for the virus; seven of them were not vaccinated at the time of infection; on the other hand, twenty had already had at least two doses.

**Fig. 2:**
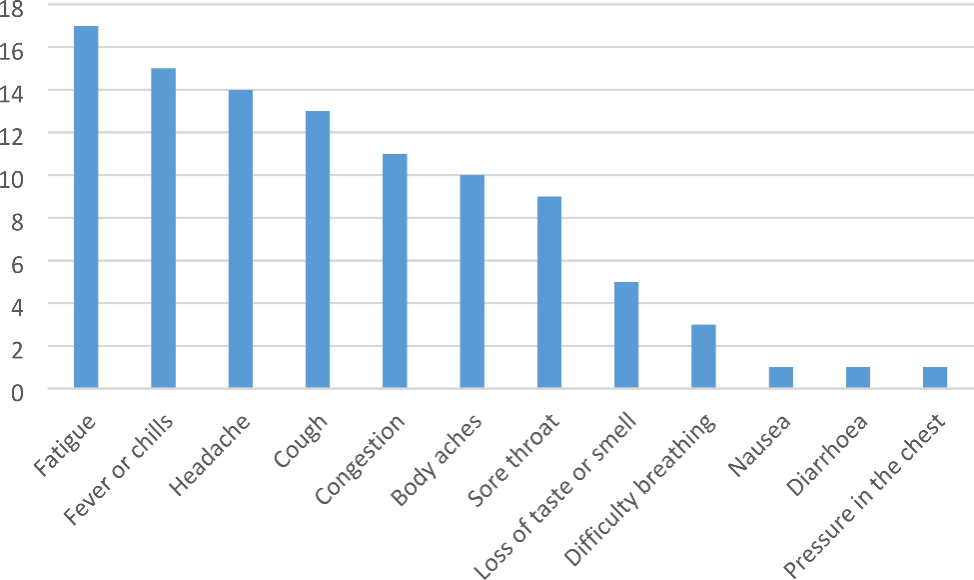
Frequency of symptoms caused by SARS-CoV-2 Infection

Figure 2 shows the symptoms reported by the patients; all were found to be mild-moderate symptoms, and no patient required hospitalization. We also reported the clinical presentation of the SARS-CoV-2 infection, stratified by IMD (Table 2).

**Fig. 3:**
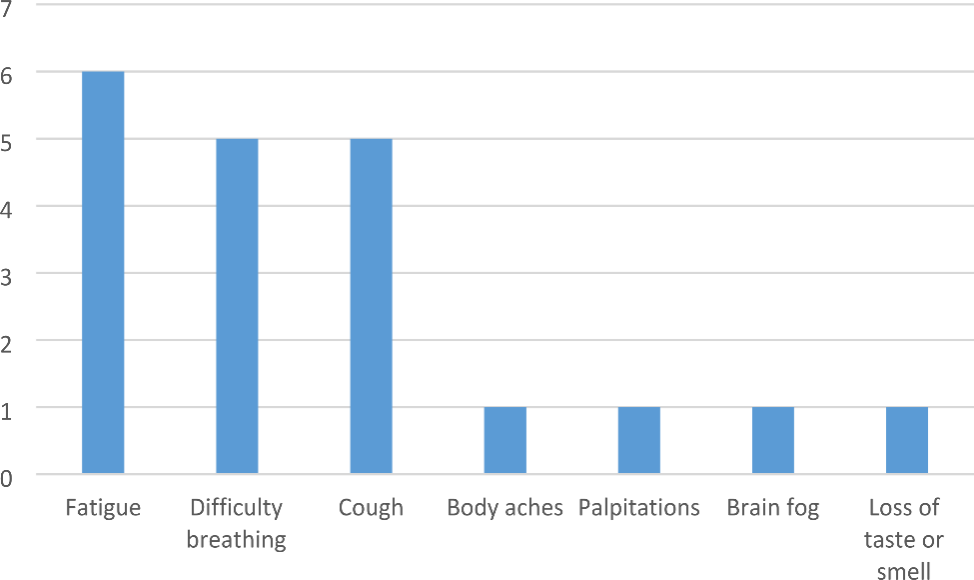
Frequency of symptoms of long-Covid

Regarding the prevalence of long-Covid, 12 patients (44%) reported symptoms that persisted after the nasopharyngeal swab test came out negative and lasted an average of 81 (± 74 SD) days. The reported symptoms are shown in Fig. 3. No patients reported headache, tingling, diarrhoea, insomnia, high temperature, rash, mood disorders, or menstrual cycle disorders. The series of patients who developed long-Covid is shown in Table 3.

**Table 2:** Symptoms reported by the patients, stratified by pathology

Metabolic Disease (number of patients who contracted SARS-CoV-2 infection /total)	Fatigue	Fever, chills	Headache	Cough	Congestion	Body aches	Sore throat	Loss of taste or smell	Difficulty breathing	Nausea	Diarrhoea	Pressure in the chest
PKU (14/38)	7/14	5/14	5/14	6/14	4/14	2/14	4/14	3/14	2/14	0/14	0/14	1/14
GSD (2/10)	1/2	2/2	2/2	0/2	1/2	1/2	1/2	0/2	0/2	0/2	0/2	0/2
GAL (3/4)	3/3	1/3	2/3	2/3	2/3	1/3	2/3	0/3	0/3	0/3	0/3	0/3
HPA (2/3)	2/2	1/2	0/2	2/2	0/2	2/2	0/2	0/2	0/2	0/2	0/2	0/2
HCU (2/3)	0/2	2/2	2/2	1/2	1/2	1/2	1/2	0/2	0/2	0/2	0/2	0/2
MTHFR (2/3)	2/2	2/2	2/2	1/2	1/2	2/2	1/2	1/2	0/2	1/2	1/2	0/2
HFI (2/3)	2/2	2/2	1/2	1/2	2/2	1/2	0/2	1/2	1/2	0/2	0/2	0/2
HT1 (0/1)	-	-	-	-	-	-	-	-	-	-	-	-
GACR (0/1)	-	-	-	-	-	-	-	-	-	-	-	-
PCD (0/1)	-	-	-	-	-	-	-	-	-	-	-	-

PKU: Phenylketonuria; GSD: Glycogen Storage Disease; GAL: Galactosemia; HPA: Hyperphenylalaninemia; HCU: Homocystinuria; MTHFR: 5,10-Methylene-tetrahydrofolate reductase deficiency; HFI: Hereditary Fructose Intolerance; HT1: Tyrosinemia type 1; GACR: Gyrate Atrophy of the Choroid; PCD: Primary Carnitine Deficiency

**Table 3:** Long-Covid manifestation, related to IMD and comorbidities

		Gastrointestinal	Endocrinological	Nephrological	Cardiovascular	Pneumological	Neurological	Duration of symptoms (days)	Fatigue	Difficulty breathing	Cough	Body aches	Palpitations	Brain fog	Loss of taste or smell	Headache	Tingling	Diarrhoea	Insomnia	Rash	Fever, chills	Mood disorder	Menstrual cycle disorders
1	PKU	N	N	N	N	N	N	42	N	Y	N	N	Y	N	N	N	N	N	N	N	N	N	N.A.
2	PKU	N	N	N	N	N	N	365	N	N	N	N	N	N	Y	N	N	N	N	N	N	N	N.A.
3	PKU	Y	N	N	N	N	N	21	N	N	Y	N	N	N	N	N	N	N	N	N	N	N	N.A.
4	PKU	N	Y	N	N	N	N	21	Y	N	Y	N	N	Y	N	N	N	N	N	N	N	N	N
5	PKU	Y	N	N	N	N	N	30	N	N	Y	N	N	N	N	N	N	N	N	N	N	N	N
6	PKU	N	N	N	N	N	N	180	N	Y	N	N	N	N	N	N	N	N	N	N	N	N	N
7	PKU	Y	Y	Y	Y	Y	N	90	Y	N	Y	N	N	N	N	N	N	N	N	N	N	N	N
8	HFI	Y	N	N	N	N	N	90	Y	N	N	N	N	N	N	N	N	N	N	N	N	N	N
9	HFI	Y	N	N	Y	N	N	42	Y	Y	N	N	N	N	N	N	N	N	N	N	N	N	N.A.
10	GAL	N	N	N	N	N	N	30	Y	N	N	N	N	N	N	N	N	N	N	N	N	N	N.A.
11	HCU	N	Y	N	N	N	N	30	Y	Y	Y	Y	N	N	N	N	N	N	N	N	N	N	N
12	GSD	Y	N	Y	N	N	N	30	N	Y	N	N	N	N	N	N	N	N	N	N	N	N	N.A.

PKU: Phenylketonuria; HFI: Hereditary Fructose Intolerance; GAL: Galactosemia; HCU: Homocystinuria; GSD: Glycogen Storage Disease; N.A.: not applicable (man or post-menopausal)

There were no statistically significant differences in BMI of patients who contracted the infection and patients who did not (25.15 vs. 25.20, p = .861), between those who had adverse reactions to the vaccine and those who did not (24.40 vs. 25.75, p = .223), between those who had long-Covid and those who did not (25.9 vs. 27.7, p = .183).

No relation between metabolic inherited disease, SARS-CoV-2 infection symptoms and adverse reaction to vaccines were observed.

Furthermore, in our cohort of 67 patients, neither baseline disease nor smoking habit was found to be associated with the contraction of infection, development of specific short- and long-term symptoms, nor the development of vaccine adverse events.

Lastly, out of 67 patients in our study, no one was treated with steroid or anti-viral therapy for SARS-CoV-2 infection, and no one needed hospitalisation.

Considering how heterogeneous our cohort of patients is, we evaluated the possibility that vaccination side effects could produce significant clinical repercussions on metabolic control. Data analysis showed that vaccination did not play a role in altering any of the parameters considered among the different parameters used to monitor each disease.

In our experience, the COVID-19 pandemic did not alter regular visits at our outpatient clinic; biochemical monitoring and imaging diagnostics proceeded according to plans.

## Discussion

The SARS-CoV-2 pandemic has had countless negative, direct and indirect public health impacts. Concerning the indirect effects, it is known that it made it harder to keep up with inpatient visits and routine procedures as scheduled. Unequivocally, it has contributed to lower quality of care towards patients with IMD, especially considering how widespread the infection was in Italy.

IMD patients are intrinsically complex; depending on their metabolic disease, various systems might be involved. For this reason, they have to attend regular visits with different specialists, they require regular metabolic testing, both in the hospital and potentially at home, and routine imaging is often necessary. Harmonious coordination of these necessary procedures, which is in itself challenging, was negatively impacted by the pandemic, making it harder to manage the underlying IMD properly. In addition to this, depending on the disease, patients may have to plan their day-to-day life around their condition, for example by following very specific diets or by the need to be regularly assisted; this reflects in a stressful burden of the disease, which impacts both the patient and the caregiver [11, 12]. Lockdown, which in Italy was adopted to contain the spread of the virus, is known to have impacted the mental health of the population [13]; it is only logical that the weight of isolation and fear of the virus had an effect at least as great in IMD patients, if not greater, considering the pre-existing conditions. Considering the aforesaid, in particular considering how frequent comorbidities in these patients are, it could be expected that SARS-CoV-2 infection might have worse outcomes compared to the general population.

Neither in the literature nor in our sample the effects of infection were more severe than those described in the general population, and the hospitalisations and deaths that occurred, described in the literature, were due to decompensation of the underlying disease due to fever or systemic inflammatory state, but not to a virus-specific effect.

Concerning the effects of SARS-CoV-2 infection, our work displays mild-moderate intensity of symptoms and absence of hospitalisation, likely due to the young age (only 9% were over 50) and low prevalence of obesity in the patients in our sample [14]. In addition to the low prevalence of obesity, our IMD patients have a low prevalence of all comorbidities which strongly correlates to negative outcomes after SARS-CoV-2 infection such as diabetes mellitus type 2, hypertension or neoplasms. We also speculate that in addition to being young, because of the IMD, our patients undergo more thorough examination compared to the general population. They undergo a regular medical examination, are evaluated at least yearly by a registered dietitian, and undergo regular complete blood testing, including their micronutrients and vitamin profiles. Our results are comparable to what has already been recently published by Mutze et al., showing that IMDs do not correlate to a more severe prognosis nor to worse outcomes [15]. Their work considered 313 adult patients with IMD from the European Registry and Network for Intoxication Type Metabolic Diseases Consortium.

Concerning the discrepancy between the severity of the infection reported by our patients and other works that have already been published [9], we attribute it to the fact that among the different kinds of inherited metabolic diseases some can incur in acute metabolic decompensation, aggravating the severity of the clinical presentation; in our sample those diseases are underrepresented. However, we need to underline that our population has a high percentage of patients who underwent at least one vaccination, which is known to yield milder symptoms [16].

Regarding SARS-CoV-2 vaccinations, these have been commercialised very rapidly, and there have been no registration studies in IMD patients. IMD patients were often considered frail patients; in Emilia-Romagna, a part of Italy, they had access to the vaccine as soon as it came out. One of the main concerns the patients had was related to the absence of specific tests on specific metabolic diseases, evaluating possible interaction with their specific condition. It should also be considered that the vaccination campaign in our region (Emilia-Romagna) prioritised vaccination for the population deemed frail, in which worse outcomes were expected in case of infection; patients with IMD were considered frail and therefore prioritised in the vaccination campaign. In our case series, 82% of patients adhered to the vaccination campaign, having already received at least two vaccine doses. Most of them received the same commercially available mRNA vaccine two or three times. A minority of patients received different types of vaccines. mRNA vaccines were administered more frequently, as according to the Italian vaccination campaign. The 18% who resulted unvaccinated did not voluntarily partake in the campaign, showing no intention of adhering to it; no patient had any medical conditions making vaccination not advised as reported on each vaccine fact sheet.

The data collected show adherence to the vaccination campaign comparable to that of the general Italian population (82% vs. 91.5% with at least one dose), with similar prevalence of doses per provider. In addition, the prevalence of SARS-CoV2 infection is also comparable to that reported by the general Italian population.

Vaccination did not result in any decompensation of the underlying disease in our cohort or in atypical adverse reaction.

Of the sample, 42% became infected with SARS-CoV2, less than 10% of these before vaccination. The most frequent symptoms reported were asthenia, fever, and headache; 19% remained asymptomatic; in no case was hospitalisation necessary or even considered. Of those who contracted the disease, 37% reported “long Covid” symptoms, the most frequent of which were shortness of breath, palpitations and fever, with an average duration of 81 (± 74) days.

Tummolo et al. recently published an interesting study evaluating 174 patients; they reported that before October 2021, 94% of the patients had received at least one dose of vaccine; unvaccinated patients were 27.1 years old on average [17]. The two main reasons were fear of the vaccine being intrinsically dangerous and fear that getting vaccinated could worsen their IMD.

With our work, we aim to reduce the percentage of unvaccinated patients, underlying how mild the adverse reactions to vaccination were and how a severe SARS-CoV-2 infection could be the cause of decompensation of the IMD.

To our knowledge this is the first work that tried to identify the impact of vaccination status on the incidence of the infection and the severity of the disease. In addition to this it is the first study that evaluates an adult cohort only.

Certainly, our study has limitations. It is not a prospective study, and a control group was not included, it was not possible to do an inferential statistical analysis.

The sample was small, especially compared to other Covid studies and heterogeneous considering the type of patients included; it evaluated patients with a wide array of different IMDs in terms of pathogenesis, clinical presentation and treatment. In addition, symptoms and adverse events were collected relying on patients’ memory of events, which sometimes dated back to the previous year. This significant lapse of time can generate the called “recall bias”. A previous work documented that the reports of the same event collected over time get progressively more discordant from the original the further away from the event itself they are collected [18].

Another potential source of biases is the limit posed by the phone call methodology itself because of the intrinsic bias of the phone call methodology in reaching evenly every member of a population; this limitation is particularly relevant considering patients who are at work and cannot take the survey [19].

In addition to this, a survey carried out by phone does not allow to consider non-verbal language and the loss of contextual data (i.e. the patient may be in an awkward place, distracting from the content of the interview or forcing rushed answers) [20].

Although telephone inquiries might have some intrinsic biases, a review by Gina Novick concluded how telephone inquiries, although disregarded by many, are handy tools for biomedical research [20].

## Conclusions

The data collected indicate adherence to the vaccination plan by patients with IMD comparable to that of the general Italian population. Adverse events to the vaccine were negligible. SARS-CoV-2 infection, which occurred in most cases after receiving at least two doses of the vaccine, did not cause serious symptoms; hospitalisation was never required. A non-negligible share of patients suffered from long-Covid symptoms.

## Methods

This is a cross-sectional, monocentric study conducted in the Clinical Nutrition and Metabolism Unit of Sant’Orsola University Hospital in Bologna, Italy, where adult patients (> 16y) with IMD have been followed since 2017.

All IMD patients (n = 93) were contacted by study staff by telephone in July 2022 to illustrate the objectives and purpose of the survey. If the patient voluntarily agreed to join the survey, information on SARS-CoV-2 infection and vaccination status was collected through a short study-specific questionnaire.

Patients had to state their SARS-CoV-2 vaccine status. If vaccinated, type and duration of adverse events of the vaccine.

In particular, patients were asked to answer yes or no regarding the following adverse events to vaccination: asthenia, injection site reaction, fever, generalized aches, headache, nausea, and diarrhoea. If the answer was yes, it was asked how many days the adverse event had lasted.

In addition, patients were also asked about SARS-CoV-2 infection, type and duration of symptoms.

Symptoms were classified as short (30 days) or long-term (more than two weeks/>30 days after SARS-CoV-2 infection).

The SARS-CoV-2 infection short symptoms investigated were classified according to the categories proposed by the WHO in 2021: asthenia, fever, headache, cough, nasal congestion, generalized pain, sore throat, loss of taste and smell, dyspnoea, nausea, diarrhoea, chest tightness.

Long-Covid investigated symptoms were: asthenia, dyspnoea, cough, generalized pain, palpitations, Covid fog, and loss of taste and smell.

The following characteristics were collected by medical records: age, gender, type of hereditary metabolic disease, and comorbidities (divided by apparatus).

Continuous variables were expressed as mean ± standard deviation (SD) or median, minimum and maximum, and IQR, depending on their distribution. Categorical variables were expressed as absolute frequency and percentage. The associations were evaluated with the chi-square test. Statistical analyses were performed using StatView for Windows 92–98 SAS Institute.

This study was approved by the local Ethics Committee (reference code EM614-2022_796/2021/Oss/AOUBo).

## Data Availability

Authors can ask the corresponding author to receive the data, after declaring the scope for this.

## Abbreviations

IMDs: Inherited Metabolic Diseases
SD: Standard Deviation
MetabERN: European Reference Network for Hereditary Metabolic Disorders
PKU: Phenylketonuria
BMI: Body Mass Index
MTHFR: Methylenetetrahydrofolate reductase
AEs: Adverse Events
